# t^4^ Workshop Report^*^: Quality Assurance of Metabolomics

**DOI:** 10.14573/altex.1509161

**Published:** 2015

**Authors:** Mounir Bouhifd, Richard Beger, Thomas Flynn, Lining Guo, Georgina Harris, Helena Hogberg, Rima Kaddurah-Daouk, Hennicke Kamp, Andre Kleensang, Alexandra Maertens, Shelly Odwin-DaCosta, David Pamies, Donald Robertson, Lena Smirnova, Jinchun Sun, Liang Zhao, Thomas Hartung

**Affiliations:** 1 Johns Hopkins University, Bloomberg School of Public Health, Center for Alternatives to Animal Testing, Baltimore, MD, USA; 2 US Food and Drug Administration, National Center for Toxicological Research, Division of Systems Biology, Jefferson, AR, USA; 3 US Food and Drug Administration, Center for Food Safety and Applied Nutrition, Laurel, MD, USA; 4 Metabolon, Inc., Durham, NC, USA; 5 Department of Psychiatry and Behavioral Sciences, Duke Institute for Brain Sciences, Duke University, Durham, NC, USA; 6 Experimental Toxicology and Ecology, BASF SE, Ludwigshafen am Rhein, Germany; 7 Bristol-Myers Squibb Pharmaceutical Co., Princeton, NJ, USA; 8 CAAT-Europe, University of Konstanz, Germany

**Keywords:** metabolomics, toxicometabolomics, quality assurance, human toxome

## Abstract

Metabolomics promises a holistic phenotypic characterization of biological responses to toxicants. This technology is based on advanced chemical analytical tools with reasonable throughput, including mass-spectroscopy and NMR. Quality assurance, however – from experimental design, sample preparation, metabolite identification, to bioinformatics data-mining – is urgently needed to assure both quality of metabolomics data and reproducibility of biological models. In contrast to microarray-based transcriptomics, where consensus on quality assurance and reporting standards has been fostered over the last two decades, quality assurance of metabolomics is only now emerging. Regulatory use in safety sciences, and even proper scientific use of these technologies, demand quality assurance. In an effort to promote this discussion, an expert workshop discussed the quality assurance needs of metabolomics.

The goals for this workshop were 1) to consider the challenges associated with metabolomics as an emerging science, with an emphasis on its application in toxicology and 2) to identify the key issues to be addressed in order to establish and implement quality assurance procedures in metabolomics-based toxicology. Consensus has still to be achieved regarding best practices to make sure sound, useful, and relevant information is derived from these new tools.

## 1 Introduction

Recent developments in safety testing regulations have initiated global changes in risk assessment. Emerging techniques like omics technologies could make toxicity testing more efficient in terms of time, cost, mechanistic understanding, and relevance to humans. Many challenges, however, need to be addressed to ensure robust and informative results sufficient for solid decision-making. Even though some omics technologies have been used for more than a decade, there is still ongoing discussion about the reproducibility of experiments and the comparability of results at different sites and on different platforms.

In Baltimore, Maryland in November 2013, the Johns Hopkins Center for Alternatives to Animal Testing (CAAT) organized a “Quality Assurance of Metabolomics” workshop with members of the NIH Research Project “Human Toxome” consortium (Bouhifd et al., 2014, 2015) together with invited experts from academia, industry, and regulatory agencies. This report highlights aspects of the presentations and discussions that took place at the workshop. It should be noted that this is not a consensus report, i.e., not every aspect of the report represents the view of all coauthors or their organizations.

Recent publications from the National Research Council, the US EPA’s (Environmental Protection Agency) computational toxicology research programs, along with the European REACH (Registration, Evaluation, Authorisation and Restriction of Chemicals) and other cosmetics legislation, are among the drivers of the current landscape changes in risk assessment and toxicity testing (Hartung, 2010, 2011). At the center of this unique advance is the conviction that emerging sciences and techniques, such as omics technologies, high-throughput screening, and computational toxicology, could make toxicity testing more efficient in terms of time, cost, animal use, and relevance to human mechanisms (Leist et al., 2008; Hartung, 2009). This conceptual framework offers many opportunities for modern toxicology, but many challenges need to be addressed to ensure sufficiently robust and informative results. The omics technologies, in particular, contribute to our understanding of toxicity mechanisms and, although some have been extensively used for more than a decade (e.g., microarrays), the reproducibility of experiments and the comparability of results at different sites and on different platforms is still subject to ongoing debate. Consensus has yet to be achieved concerning best practices in many critical topics, such as the experimental design and protocols for sample preparation and handling, data processing, statistical analysis, and interpretation. One major challenge is how to ensure that sound, useful and relevant information is derived from these new tools. Quality assurance is the first response. The diversity of the technological platforms, complexity of biological systems, and variety of analytical and computational methods make it critical to adopt measures and procedures for ensuring the quality of the data.

Metabolomics, an interdisciplinary science that combines analytical chemistry, biochemistry, statistics, and bioinformatics, is one of the most promising omics tools in the post-genome era. It is primarily the comparative analysis of the endogenous metabolites present in any biological system at a given physiological state. Metabolomics also includes aspects of patho-biochemistry, systems biology, and molecular diagnostics when applied to toxicology (Griffiths et al., 2010). Its approaches have been applied in clinical settings and have been increasingly expanded to other fields (such as toxicology), because they have the ability to provide information that allows to better understand the mechanisms of toxicity (Craig et al., 2006; Heijne et al., 2005; Ruepp et al., 2002; Schnackenberg et al., 2006, 2009; Montoya et al., 2014). From an analytical perspective, the goal of metabolomics in toxicology studies is to “*achieve a comprehensive measurement of the metabolome and how it changes in response to stressors, with biological payoff being an illumination of the relationship between the perturbations and affected biochemical pathways*” (Robertson and Lindon, 2005). Toxicological applications have been detailed in many publications (Ramirez et al., 2013; Bouhifd et al., 2013; Robertson, 2005). In early 2000, metabolomics was suggested for the first time as a new technique for rapid toxicity screening (Robertson and Bulera, 2000), was used in academic research (to predict liver and kidney toxicity *in vivo*) Lindon et al., 2005)), and also in industry to elucidate toxicological modes of action allowing for early safety decisions and lowering the cost through reduced animal studies (van Ravenzwaay et al., 2012). *In vitro* applications are emerging and have been driven by two major factors: 1) the call for a better understanding of biochemical changes induced by a toxic insult in a defined and controllable experimental system and 2) the increasing requirement to move towards the use of human-relevant, non-animal alternatives (Ramirez et al., 2013). *In vitro* measurements of intracellular metabolites have allowed for organ-specific *in vitro* toxicity testing, e.g., neurotoxicity (van Vliet et al., 2008), renal toxicity (Ellis et al., 2011), hepatotoxicity (Ruiz-Aracama et al., 2011), mitochondrial toxicity (Balcke et al., 2011), and lung toxicity (Vulimiri et al., 2009).

Undoubtedly, the promise of metabolomics in various scientific disciplines, including *in vitro* toxicology, is recognized. Nevertheless, many obstacles must be addressed before the discipline can achieve its full potential. Besides the challenges inherent to any toxicological study, we discussed the issues specific to metabolomics with an emphasis on *in vitro* applications. These included quality assurance practices in academia and regulatory agencies and also aspects of conducting metabolomics studies in industrial settings.

## 2 Quality assurance in toxicology studies

Quality assurance is fundamental to all good scientific practice. The maintenance of high standards is essential for ensuring the reproducibility, reliability, credibility, acceptance, and proper application of the results generated. The challenges and limitations of models and test methods in toxicology have been recognized and discussed (Hartung, 2009, 2011, 2013). Currently, toxicological risk assessments rely mainly on *in vivo* animal experimentation that is often expensive and follows test guidelines that are usually a few decades old. (However, by adding omics measurements to such studies, the information content and therefore scientific quality of such *in vivo* studies can be significantly increased). The throughput is low, preventing many substances from being adequately assessed (Grandjean and Landrigan, 2006; Judson et al., 2009). Selecting a test species that will best predict the human response is also challenging. On the other hand, *in vitro* toxicology studies depend significantly on cell models that differ in many aspects from normal physiology, making them difficult to reproduce in culture (Hartung, 2007a). Besides these intrinsic challenges, the discipline suffers from a lack of standards in methods and model standardization, and inefficient documentation and reporting.

Despite these problems, guidance has been developed that acknowledges the inherent variation of *in vitro* test systems and promotes standardization. Good Cell Culture Practice (GCCP) sets the minimum standards for any *in vitro* work involving cell and tissue cultures (Hartung et al., 2002). It aims to reduce uncertainty in the development and application of *in vitro* procedures by encouraging the establishment of principles for greater international harmonization, standardization, and rational implementation of laboratory practices, quality control systems, safety procedures, and reporting (Coecke et al., 2005). This guidance is comparable to the OECD Principles of Good Laboratory Practice (GLP) (OECD, 2004) (the two have actually cross-fertilized each other), which cannot normally be fully implemented in basic research because of cost and lack of flexibility. The requirement that all personnel need to be fully trained before executing tests, in particular, cannot be met in an academic setting, where much of the work is done by students. However, through some simple actions based on GLP principles, a higher level of quality can be achieved even in academic research.

Quality assurance of *in vitro* methods could be further reinforced by the principles of validation. The term ‘validation’ is used differently in different contexts. All fields of science and engineering technically validate methods with regard to the internal performance parameters of a method. Formal validation was introduced for the acceptance of regulatory test methods to help agencies decide on the implementation of new tools, especially those replacing animal experiments. Predictivity, usually in comparison to the traditional (animal) test method, is also validated in addition to the internal performance characteristics (reliability). Guidelines were primarily developed by three organizations: the Organisation for Economic Cooperation and Development (OECD) (OECD, 2005), the European Centre for the Validation of Alternative Methods (ECVAM) (Hartung et al., 2004), and the Interagency Coordinating Committee on the Validation of Alternative Methods (ICCVAM). Criteria to be addressed in a validation exercise include: test definition (including purpose, need, and scientific basis), relevance of the test method, repeatability and reproducibility, inter-laboratory transferability, predictive capacity, and applicability domain. Questions arise about whether the validation process, as it has been formalized over the last two decades, might meet the challenges of emerging methods and technologies (such as omics), especially in toxicity testing (Hartung, 2007b; Leist et al., 2012).

A pragmatic approach would adapt some of the principles and criteria listed above for ensuring some degree of quality that could lay the ground for a quality assurance system in toxicometabolomics studies. In other words, toxicometabolomics needs quality-controlled model systems, and simply adding an omics endpoint does not make a test better.

## 3 Toxicometabolomics quality assurance

The comprehensive analysis of small molecules and their changes in response to stressors is a challenging exercise. The success of a toxicometabolomics study often depends on multiple experimental, analytical, and computational steps. A typical workflow used in metabolomics studies is outlined in Table 1. This process involves many steps, starting from the actual study design, which depends on the adopted metabolomic approach. Indeed, many approaches are currently used in metabolomics studies ranging from fingerprinting to non-targeted profiling to targeted analysis (Robertson et al., 2011). The study design involves the selection of the test system (e.g., animal model, *in vitro* cell culture), the type of the stressor, and the route of exposure. The choice of the biological matrix is also important; typical matrices analyzed include blood, serum, and urine, as well as intra- and extra-cellular extracts. The extraction method has to be specifically developed and optimized for each matrix before sample preparation and analysis. Once the metabolite data are generated, they are handled in order to prepare and reduce analytical instrument raw data (e.g., MS chromatograms) to data matrices for further analysis. This typically involves the execution of a series of tasks ranging from low-level processing (background correction, feature detection, normalization, alignment, etc.) to higher level processing consisting of various tools and methods for interpretation and visualization of the pre-processed data.

In his presentation during the workshop, Dr Donald Robertson stated, “*In the past fifteen years, I have been involved in approximately 500 metabolomic studies. Of those studies that failed, more than 90% of the failures could be attributed to errors in study design, study conduct, sample collection, or sample preparation. Relatively few failed due to analytical reasons*.” Furthermore, the American Society for Mass Spectrometry (ASMS) survey of about 600 participants at its 2009 conference (American Society for Mass Spectrometry, 2009) clearly showed that the analytical element was considered of less concern than the interpretation of metabolomics data and its biological significance. We will summarize below the main elements of a metabolomics study in toxicology and related quality measures.

## 4 Study design

The suitable design of scientific studies is the first necessary condition to ensure robust and trustworthy conclusions. By definition, experimental design is the process of planning data gathering in order to meet predefined objectives and answer the research question of interest as clearly as possible. Experimental design takes into account specific considerations for the experiment type (e.g., treatment vs. control), experimental variables (e.g., dose response, time dependence), experiment controls, and acceptance criteria. The number of replicates considered in the study is a critical determinant of the quality of the experiment. There is no “magic” number relative to the number of replicates needed, since this will depend on the multiple sources of variability in the experiment. The Metabolomics Standards Initiative (MSI) (Sumner et al., 2007) suggests a minimum of three to five replicates, with a preference of biological replication (i.e., repetitive analyses of different samples obtained under the same experimental conditions) over technical replication (repetitive analyses of the same sample). It is a good practice, however, to conduct a preliminary pilot study to evaluate the data variation under the specific conditions and to perform a power analysis to guide the determination of the optimal number of replicates. Traditional power analysis would calculate the number of replicates based on the expected effect strength, the significance level aimed for, and the variability of the measurement in the model. This is not as easy for metabolomics, as effect strengths are typically small, multiple parallel measurements have to be accounted for (false discovery rate corrections), and the very different variability for different metabolites impair such calculations. Therefore, extensive evaluation of (control) variability over time is needed to a) ensure reproducibility and robustness, and therefore reliability of the measurements and b) enable the identification of biologically significant results. For the latter, statistically significant changes occurring in an experiment can be compared to the historical control data and variability of the respective metabolite (van Ravenzwaay et al., in press). In addition, the use of quality control (QC) samples is recommended and has been increasingly adopted (Dunn et al., 2012). These are usually representative of all the samples being analyzed in the study to represent a “mean” of all analyzed metabolites (Gika et al., 2007). A QC sample in the context of metabolomics could be obtained by pooling all samples in the study or by using additional control groups and pooling the samples derived from these control groups. Aliquots of a unique “pooled” QC sample, applied for an entire study at regular intervals, can help determine variations of all processes involved in terms of data acquisition (e.g., retention time and abundance) and also in data pre-processing (e.g., feature extraction). Furthermore, blank samples, which are analyte-free and prepared exactly as the test samples, give an idea of the overall levels of contamination and carryover. An additional quality measure in the experimental design is randomization of the sample analysis sequence. This procedure minimizes the bias introduced when preparing and analyzing replicate samples jointly. According to our own experience, QC samples account for about 30% and up to 50% of the total number of injections in an LC-MS run.

## 5 Sampling and extraction

Differences in methods to collect, prepare, store, and otherwise handle samples are important sources of bias in life sciences in general and have been a major problem in biomarker detection as, for example, noted by Teahan et al. (2006), where allegedly promising results were sometimes difficult to reproduce and validate. Diverse biological systems, such as microorganisms, plants, biofluids or mammalian cells, are studied in metabolomics, making it challenging to devise a unique method or guideline for sample collection and preparation.

There are rather general good practices and examples, such as those included in the NCI best practices for biospecimen resources (National Cancer Institute, 2011). Although the guideline is primarily intended for human specimens, it provides technical and operational best practices to ensure levels of consistency and standardization. It also identifies a variety of factors that may affect biospecimen quality and thus research results. Recommendations include, where possible, the use of validated methods, training of technical staff, inclusion of appropriate quality control and reference samples, randomization, and standardized methods for documenting. Guidelines for the use of biofluids in proteomics studies (Rai et al., 2005) are also applicable to metabolomics. They evaluate a number of pre-analytical variables that can potentially impact the outcome. These include, among others, the sample type, the collection system, the processing methods, and storage parameters. During the workshop, Dr Hennicke Kamp reported that standardization of all steps, from collecting the sample from a biological system through sample preparation and metabolite extraction is essential to obtaining robust and reliable metabolome data. The large diversity of physico-chemical properties of the metabolome poses an additional challenge. Chemical compounds analyzed differ in molecular weight, polarity, boiling and melting point, functional groups, etc. Moreover, these compounds are present in concentrations that span orders of magnitude within the same sample (Maier et al., 2010).

Metabolomics involves, therefore, the analysis of a heterogeneous chemical space and across a broad dynamic range, which makes considerations for standardization of protocols challenging. An efficient method would allow the adequate recovery of the largest number of metabolites from samples while preventing the exclusion of compounds due to their physical or chemical properties (Winder et al., 2008). While it is obvious that no unique analytical method can fulfill these requirements, consistent quenching, extraction protocols, as well as adequate sample storage would limit variability in metabolite extraction and analysis (Zhou et al., 2012). Documentation in the form of Standard Operating Procedures (SOPs), optimized for the specific metabolomic application, should be detailed enough to allow an unambiguous and reproducible execution of the procedure (Bouhifd et al., 2013).

## 6 Metabolomics data complexity

A fundamental characteristic of metabolomics is the huge diversity of chemicals involved. Unlike a genome, which involves only four bases, and the proteome with its twenty amino acids, the metabolome consists of at least a few thousand chemicals (Wishart, 2011). The various chemical and physical properties of these molecules would require a combination of analytical technologies to obtain good coverage. Historically, the analytical tools of choice in metabolomics have been NMR and MS. The latter is combined with a chromatographic separation technique such as liquid chromatography (LC) or gas chromatography (GC). The characteristics, advantages, limitations, and differences between the technologies and platforms have been extensively described in several review articles and will not be addressed here (Kaddurah-Daouk et al., 2008; Robertson, 2005; Dunn and Ellis, 2005).

Despite the recent technology advances, no single analytical platform is a perfect tool for metabolomics, with all having advantages and limitations, although LC-MS now appears to be the preferred technology in many studies. Besides the biological variability described earlier, metabolomics data can suffer from analytical variability. This includes mainly drifts in retention times, altered instrument sensitivity, and – very rarely – drifts in measured mass to charge ratio (m/z) values. Although the technologies involved are complex, it is well accepted in the community that the analytical process is not the main limitation.

One of the biggest challenges in metabolomics remains metabolite identification. An accurate identification of the chemicals involved in any particular study is necessary to derive meaningful biological information. It is now a very common practice to generate metabolomic datasets comprising thousands of “features,” but their identification is certainly not straightforward. A mass spectrometry measurement typically results in a list of entities represented by mass-to-charge (m/z) ratio, retention time (RT), and intensity. These parameters might be informative but do not provide a direct chemical annotation of the entity in question. One needs first to convert the raw analytical data to metabolites (namely chemicals).

The accuracy and confidence in this conversion (identification, in other words) vary widely because of the complexity of the process and its dependence on the analytical platform and robustness of the methods applied, as well as the databases and resources used (Creek et al., 2014). Indeed, this process should discriminate not only between metabolites with different masses, but also those with the same nominal mass but different molecular formula and monoisotopic mass, and also metabolites with the same nominal and monoisotopic masses but different chemical structures. In addition, a single metabolite can form multiple different ion types (in the case of electrospray ionization, for example) such as sodium and potassium adducts, along with the standard protonated form (Dunn et al., 2013). Diverse strategies have been adopted with different levels of confidence. These confidence levels were divided into four categories by a dedicated working group of the Metabolomics Standards Initiative (MSI) and the following definitions were proposed (Sumner et al., 2007):

1. Confidently identified compounds where at least two orthogonal properties (e.g., m/z, RT, fragmentation mass spectrum) of the candidate metabolite are verified with an authentic reference standard under the same analytical conditions;
2. Putatively annotated compounds where the physicochemical properties are compared to chemical library without reference to authentic standards;
3. Putatively characterized compound classes based upon characteristic physicochemical properties of a chemical class of compounds (e.g., lipids), or by spectral similarity to known compounds of a chemical class, and;
4. Unknown compounds which are unidentified and unclassified metabolites that can still be differentiated using spectral data.

Although the exact basis for what constitutes valid metabolite identification could be debated, a major contribution of the MSI is the detailed formulation of the reporting needs of the identification procedure and its performance. Different strategies could be adopted, but metabolites are typically characterized on the basis of accurate mass, retention time, and tandem mass spectrometry (MS/MS) data. First, m/z values are searched in metabolite databases (peak annotation). When a hit is returned within the expected error of the mass spectrometer, the annotation is still putative and sometimes needs manual curation. To increase the level of confidence as described above, an authentic reference standard is used, and retention time and/or MS/MS data is generated in the same analytical conditions and compared to that from the biological sample (Patti et al., 2012). Following this description, two important elements emerge regarding quality assurance – namely, metabolite databases and reference standards.

Databases (DBs) are sources of chemical information in the form of web-based or locally hosted services, and fulfill many objectives. They are of different types and contain diverse information such as metabolic pathway information, compound-specific information, spectral information, disease/physiology information, or organism-specific metabolomic information (Wishart et al., 2009). Although these resources are increasingly helpful, some limitations still exist. DBs have different coverages, and although they might be partly complementary, missing metabolite identifiers and ambiguous names for metabolites affect the comparison (Stobbe et al., 2011). Considerable manual intervention and curation is required to unify the DBs, prompting a need for standardization of the metabolite names and identifiers. This effort is challenging since it needs not only expert knowledge but also some degree of automation.

## 7 Metabolomics in a systems biology context

Systems biology has been defined as the “*study of the mechanisms underlying complex biological processes as integrated systems of many diverse, interacting components. It involves (1) a collection of large sets of experimental data (by high-throughput technologies and/or by mining the literature of molecular biology and biochemistry); (2) proposal of mathematical models that might account for at least some significant aspects of this data set; (3) accurate computer solution of the mathematical equations to obtain numerical predictions; and (4) assessment of the quality of the model by comparing numerical simulations with the experimental data*” (Ferrario et al., 2014). Metabolomics is a key tool for producing such large datasets and lends itself especially to the characterization of phenotypic changes in a systems toxicology approach (Hartung et al., 2012). The major advantage of toxicology compared to other disciplines is that we have the “disease agent” at hand, i.e., we can experimentally induce and monitor pathogenesis and are not restricted to comparison of healthy versus diseased tissue and biofluids. The mathematical models of metabolism, however, will have to reflect the dynamics of the networked systems and will likely depend on the measurement of metabolite fluxes. This topic will open up further aspects of quality assurance beyond what was discussed in this workshop. The Human Toxome project is pioneering some of this (Bouhifd et al., 2015) and not surprisingly prompted the need for discussion about quality assurance via this workshop.

## 8 Future directions: Need for collaborative activities

The discussions of this workshop showed that a combination of expert consensus – for example, reporting standards and good practices – and experimental assessments (e.g., ring trials between laboratories) is needed. The importance of both analytical and biological validation was emphasized. Validation in a broad sense demonstrates suitability for an intended purpose or “fitness for purpose.” To simplify, we may distinguish two main components: reliability (robustness/quality/confidence) and relevance (usefulness/biological utility). These two elements, if satisfied, will ensure reproducible and meaningful research. Such discussions took place for transcriptomics a decade ago (for example, the first transatlantic consensus workshop on validation of transcriptomics (Corvi et al., 2006)).

The present workshop was prompted by the ongoing Human Toxome project (Bouhifd et al., 2013, 2014). This project aims for the identification of pathways of toxicity (Kleensang et al., 2014) by using a multi-omics approach (Hartung and McBride, 2011). For the purpose of this project, it will be necessary to assess especially whether intracellular metabolomics, i.e., the metabolomic analyses of cell extracts, are sufficiently robust to allow reliable and consistent identification of specific changes in newly produced or altered metabolites in response to a known toxicant stimulus. This is a prerequisite for unambiguous identification of the underlying molecular mechanisms or pathways of toxicity. The overall strategy would consist of generating standardized biological samples and assessing within-run, within-lab, and between-lab reproducibility of metabolomics analysis. Unfortunately, such quality assurance studies lack appeal for most funding bodies, but they have the potential not only to move forward a given project but to further the proper use of an entire technology.

## Figures and Tables

**Tab. 1:** Typical metabolomics workflow

**Study design**	Problem formulation Experimental condition definition Toxicant treatment (time, route, formulation, etc.)
**Sample preparation**	Harvesting/sampling/preparing/storage Metabolite extraction
**Data generation**	Measurement (e.g., LC-MS) Data processing Feature selection
**Confirmation**	Metabolite identification Metabolite quantification
**Conclusions (biological relevance)**	Modeling Data interpretation Hypothesis generation and/or verification Reporting
